# Linseed Components Are More Effective Than Whole Linseed in Reversing Diet-Induced Metabolic Syndrome in Rats

**DOI:** 10.3390/nu11071677

**Published:** 2019-07-22

**Authors:** Siti Raihanah Shafie, Stephen Wanyonyi, Sunil K. Panchal, Lindsay Brown

**Affiliations:** 1 Functional Foods Research Group, University of Southern Queensland, Toowoomba, QLD 4350, Australia; 2 School of Health and Wellbeing, University of Southern Queensland, Toowoomba, QLD 4350, Australia

**Keywords:** linseed, secoisolariciresinol diglucoside, obesity, blood pressure, high-carbohydrate, high-fat diet

## Abstract

Linseed is a dietary source of plant-based ω–3 fatty acids along with fiber as well as lignans including secoisolariciresinol diglucoside (SDG). We investigated the reversal of signs of metabolic syndrome following addition of whole linseed (5%), defatted linseed (3%), or SDG (0.03%) to either a high-carbohydrate, high-fat or corn starch diet for rats for the final eight weeks of a 16–week protocol. All interventions reduced plasma insulin, systolic blood pressure, inflammatory cell infiltration in heart, ventricular collagen deposition, and diastolic stiffness but had no effect on plasma total cholesterol, nonesterified fatty acids, or triglycerides. Whole linseed did not change the body weight or abdominal fat in obese rats while SDG and defatted linseed decreased abdominal fat and defatted linseed increased lean mass. Defatted linseed and SDG, but not whole linseed, improved heart and liver structure, decreased fat vacuoles in liver, and decreased plasma leptin concentrations. These results show that the individual components of linseed produce greater potential therapeutic responses in rats with metabolic syndrome than whole linseed. We suggest that the reduced responses indicate reduced oral bioavailability of the whole seeds compared to the components.

## 1. Introduction

Linseed or flax (*Linum usitatissimum* L.) has widely reported health benefits from studies with many forms including whole or ground seeds, oil, defatted meal, and mucilage extracts [1,2]. Linseed and its components, especially *α*–linolenic acid (ALA, C18:3*n*–3) and the lignan, secoisolariciresinol diglucoside (SDG), may protect against metabolic syndrome and cardiovascular disease by lowering blood pressure, reducing blood glucose concentrations, delaying postprandial glucose absorption, and decreasing oxidative stress and inflammation [3,4,5]. However, the health benefits of introducing linseed into the diet have not been fully defined [6]. In addition, processing including dehusking, crushing, milling, and defatting may increase bioavailability of individual components such as lignans and ALA [7,8,9]. Furthermore, no studies have compared physiological responses to whole linseed or linseed components using the same animal model or humans.

Linseed has a hard outer layer which may allow the seeds to pass unchanged through the gut and reduce absorption of useful nutrients by the body [10]. Thus, it may be more beneficial to consume ground linseeds over whole linseeds. This implied difference in oral bioavailability could markedly alter the choice of linseed preparations as functional foods, since both whole linseeds and ground linseed flour are readily available. In humans fed muffins with either 30 g whole or ground linseed, or flaxseed oil with 6 g ALA, plasma ALA concentrations were 0.024 mg/mL with whole linseed (not significantly different from control) but increased to 0.031 mg/mL with ground linseed and 0.055 mg/mL with linseed oil, suggesting reduced absorption from whole linseeds [11]. In a randomized, crossover study involving 12 healthy subjects, the bioavailability of enterolignans formed as lignan metabolites in the liver more than tripled after feeding on crushed linseed relative to whole linseed and further increased with milled linseed [8]. In rats following oral administration, SDG was metabolized in the gastrointestinal tract and not absorbed while the oral bioavailability of secoisolariciresinol was about 25% with a half–life within the body following intravenous administration of 4 h [12].

In this study, we evaluated the cardiovascular, liver, and metabolic responses of whole linseed, and two of its components, defatted ground linseed, and SDG–enriched fraction, by using an established model of high-carbohydrate, high-fat diet-fed rats mimicking the human metabolic syndrome [13]. We have compared these results with our earlier study on 3% linseed oil containing ALA, which normalized systolic blood pressure, and improved heart function and glucose tolerance [14]. Measurements included body weight, systolic blood pressure, oral glucose tolerance test, left ventricular diastolic stiffness, histology of the heart and liver, and plasma biochemistry. Doses of the linseed components were chosen so as to be similar to the proportion in whole linseed. Our hypothesis was that whole linseeds and the isolated components would improve cardiovascular, metabolic, and liver changes in diet-induced metabolic syndrome in rats.

## 2. Materials and Methods

### 2.1. Rats and Diet

All experimental protocols were approved by the University of Southern Queensland Animal Ethics Committee under the guidelines of the National Health and Medical Research Council of Australia. Male Wistar rats were purchased from Animal Resource Centre, Murdoch, WA, Australia. Rats were housed individually in temperature-controlled, 12 h light/dark conditions in the animal house facility of the University of Southern Queensland. The rats were acclimatized and given free access to water and standard rat powdered food prior to initiation of the protocol diets.

Rats (8–9 weeks old, weighing 330–340 g, *n* = 96) were randomly divided into 8 experimental groups: corn starch diet-fed rats (C; *n* = 12), corn starch diet-fed rats treated with 5% whole linseed in food (CW; *n* = 12), corn starch diet-fed rats treated with 3% defatted ground linseed in food (CD; *n* = 12), corn starch diet-fed rats treated with 0.03% SDG in food (CS; *n* = 12), high-carbohydrate, high-fat diet-fed rats (H; *n* = 12), high-carbohydrate, high-fat diet-fed rats treated with 5% whole linseed in food (HW; *n* = 12), high-carbohydrate, high-fat diet-fed rats treated with 3% defatted ground linseed in food (HD; *n* = 12), and high-carbohydrate, high-fat diet-fed rats treated with 0.03% SDG in food (HS; *n* = 12). Preparation of C and H diets has been described previously [13]. The energy densities of C and H diets were 11.23 kJ/g and 17.83 kJ/g, respectively, with an additional 3.85 kJ/mL in drinking water for fructose intake in high-carbohydrate, high-fat diet-fed rats [13].

Whole linseed- and defatted linseed–supplemented diets were prepared by replacing 5% water with 5% whole linseed (not ground) and 3% water with 3% defatted linseed, respectively, in C and H diets. The whole linseed dose replicated our previous study which used 5% chia seeds in food [15], as the oil composition of chia seed and linseed are similar. Since the oil content of linseed is about 40% [16], the non-oil component, defined here as defatted linseed, is 60% so defatted linseed flour was added at 3% in the food. The SDG-supplemented diets were prepared by adding 0.03% SDG (0.3 g of SDG/kg food) in C and H diets.

The whole linseed, defatted linseed, and SDG diets were administered for 8 weeks starting 8 weeks after the initiation of C or H diet. H, HW, HD, and HS groups were given 25% fructose in drinking water along with the diets for the 16-week duration of the study. Normal drinking water without any supplementation was given to C, CW, CD, and CS rats. Rats were monitored daily for body weight and food and water intakes. Energy intake and food conversion efficiency were calculated based on the food intake and body weight gain [14,15].

Whole linseed was a gift from AustGrains (Moree, NSW, Australia) and was also ground and extracted with *n*-hexane to produce defatted linseed. SDG (40% purity) was a gift from the Archer Daniels Midland Company (Chicago, IL, USA). The analysis of SDG content was conducted by St. Boniface Hospital Research, Winnipeg, MB, Canada. Total gross energy content of whole linseed, defatted linseed, and SDG samples were measured by bomb calorimetry (XRY-1A Oxygen Bomb calorimeter, Shanghai Changji Geological Instrument Co. Ltd., Shanghai, China) in triplicate. One gram of whole linseed, defatted linseed, or SDG were burnt in compressed oxygen (25 kg/cm^2^) in the calorimetric bomb immersed in water. The energy densities for whole linseed, defatted linseed, and SDG were 23.76 kJ/g, 16.98 kJ/g, and 17.48 kJ/g, respectively.

### 2.2. Measurements in Live Rats

Systolic blood pressure was measured at the end of the protocol under light sedation by intraperitoneal injection with Zoletil (tiletamine 10 mg/kg, zolazepam 10 mg/kg; Virbac, Peakhurst, NSW, Australia). Measurements were performed using an MLT1010 Piezo-Electric Pulse Transducer (ADInstruments, Sydney, NSW, Australia) and an inflatable tail-cuff connected to an MLT844 Physiological Pressure Transducer (ADInstruments) connected to a PowerLab data acquisition unit (ADInstruments) [13].

Oral glucose tolerance tests were performed at the end of the protocol on rats after overnight (12 h) food deprivation. During this time, fructose-supplemented drinking water in H, HW, HD, and HS rats was replaced with tap water. Basal blood glucose concentrations were determined in tail vein blood using Medisense Precision Q.I.D. glucometer (Abbott Laboratories, Bedford, MA, USA). The rats were given 2 g/kg body weight of glucose as a 40% (w/v) aqueous glucose solution via oral gavage. Tail vein blood samples were taken at 30, 60, 90, and 120 min following glucose administration [13].

Dual-energy X-ray absorptiometry (DXA) was performed on all rats after 16 weeks of feeding using a Norland XR36 DXA instrument (Norland Corp., Fort Atkinson, WI, USA). Rats were anesthetized using intraperitoneal injection of Zoletil (tiletamine 10 mg/kg and zolazepam 10 mg/kg) and Ilium Xylazil (xylazine 6 mg/kg; Troy Laboratories, Smithfield, NSW, Australia). Scans were analyzed using the manufacturer’s recommended software for use in laboratory animals (Small Subject Analysis Software, version 2.5.3/1.3.1; Norland Corp.) [13]. Visceral adiposity index (%) was calculated based on the abdominal fat content obtained during terminal experiments [15].

### 2.3. Measurements after Euthanasia

Terminal euthanasia was induced by intraperitoneal injection of Lethabarb (pentobarbitone sodium, 100 mg/kg; Virbac) and ~6 mL blood was immediately drawn from the abdominal aorta, collected into heparinized tubes, and centrifuged for plasma [13]. Hearts (*n* = 8–10) were separated into right ventricle and left ventricle with septum for weighing. Liver and abdominal fat pads (retroperitoneal, epididymal, and omental) were isolated and weighed (*n* = 8–10). Organ weights were normalized to the tibial length and presented in mg of tissue/mm of tibial length [13].

A portion of the heart, liver, small intestine, and large intestine was collected and fixed in 10% neutral buffered formalin for 3 days. Standard histological procedures were followed to process tissues for staining with hematoxylin and eosin or picrosirius red staining [13]. Two slides were prepared per tissue specimen and two random, non-overlapping fields per slide were taken to avoid biased analysis. To examine collagen distribution in the heart, the tissue was stained with picrosirius red stain and imaged using EVOS FL Color Imaging System (version 1.4 (Rev 26059); Advanced Microscopy Group, Bothwell, WA, USA) [14]. Small and large intestine sections were stained with periodic acid-Schiff stain to identify goblet cells [17]. Left ventricular collagen deposition was estimated by analysis with NIH ImageJ software (https://imagej.nih.gov/ij/).

Plasma samples collected during terminal experiments were used to test plasma activities of alanine transaminase and aspartate transaminase, and plasma concentrations of total cholesterol, triglycerides, and nonesterified fatty acids [13].

### 2.4. Statistical Analysis

All data are presented as mean ± standard error of the mean (SEM). Group data were tested for variance using Bartlett’s test. Variables that were not normally distributed were transformed (using log 10 function) prior to statistical analysis. Groups were tested for effects of diet, treatment, and their interactions using two-way analysis of variance. When interaction and/or the main effects were significant, means were compared using Newman-Keuls multiple-comparison post hoc test. All statistical analyses were performed using Prism version 6.00 for Windows (GraphPad Software, San Diego, CA, USA). *p*-value of < 0.05 was considered as statistically significant.

## 3. Results

### 3.1. Dietary Intakes

Food and water intakes were lower in H rats than in C rats but energy intakes were higher in H rats than in C rats (Table 1). There were no differences between food intakes of C, CW, CD, and CS or between H, HW, HD, and HS groups (Table 1). Water intake was unchanged among C, CW, CD, and CS groups and there was no difference in water intake among H, HW, HD, and HS rats (Table 1). Doses of SDG were 31.9 ± 1.3 mg/kg/day and 15.9 ± 0.3 mg/kg/day for CS and HS rats, respectively. Intakes of whole linseed were 4.36 ± 0.14 g/kg/day and 2.51 ± 0.16 g/kg/day for CW and HW rats, respectively, while intakes of defatted linseed were 2.64 ± 0.09 g/kg/day and 1.57 ± 0.04 g/kg/day for CD and HD rats, respectively.

### 3.2. Body Composition and Organ Weights

**Figure 1:**
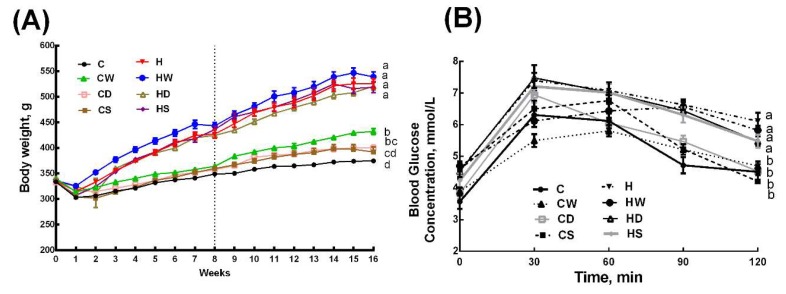
Effects of whole linseed, defatted linseed, and secoisolariciresinol diglucoside (SDG) on (**A**) body weight and (**B**) oral glucose tolerance test. The vertical dotted grid line in (**A**) at week 8 represents the start of treatment for rats. Data are presented as mean ± SEM, *n* = 10–12. End-point means with different letters (a, b, c, or d) are significantly different, *p* < 0.05. C, corn starch diet-fed rats; CW, corn starch diet-fed rats treated with whole linseed; CD, corn starch diet-fed rats treated with defatted linseed; CS, corn starch diet-fed rats treated with SDG; H, high-carbohydrate, high-fat diet-fed rats; HW, high-carbohydrate, high-fat diet-fed rats treated with whole linseed; HD, high-carbohydrate, high-fat diet-fed rats treated with defatted linseed; HS, high-carbohydrate, high-fat diet-fed rats treated with SDG.

Body weight was higher in H rats than in C rats and whole linseed, defatted linseed and SDG did not change body weight in HW, HD, and HS rats, whereas the body weight was higher in CW rats than in C rats, and CS and CD rats had intermediate body weights to C and CW rats (Figure 1A and Table 1). Body weight gain was lower in HS rats compared to H, HW, and HD rats, whereas body weight gain was in the order C=CS>CD>CW among the C diet groups (Table 1). Feed conversion efficiency was higher in H rats than in C rats. Interventions did not change feed conversion efficiency in H diet-fed rats (HW, HD, and HS rats), whereas whole linseed increased the feed conversion efficiency in CW rats with no change in CS and CD rats (Table 1). Bone mineral content was higher in H rats than in C rats. None of the interventions changed bone mineral content in C diet groups (CW, CD, and CS), whereas HD rats showed reduction in this parameter compared to H, HW, and HS rats (Table 1). Lean mass did not differ between C and H rats. CW, CD, and HD rats had higher lean mass; CS, H, and HS rats had lower lean mass, whereas C and HW had intermediate lean mass (Table 1). H rats had higher fat mass compared to all C diet groups (C, CW, CD, and CS rats). HD rats had lower fat mass compared to H and HW rats, whereas HS rats had fat mass intermediate to H and HD rats (Table 1). Abdominal circumference and visceral adiposity index were unchanged in CW, CD, and CS rats compared to C rats, whereas these parameters were increased in H rats compared to C rats (Table 1). HD rats had lower abdominal circumference compared to H and HW rats, whereas visceral adiposity index was higher in HW rats compared to HS and HD rats (Table 1).

Retroperitoneal, epididymal, and omental fat pads were higher in H rats than in C rats. CW, CD, and CS rats had no difference in these fat pads and total abdominal fat compared to C rats. SDG decreased epididymal fat in HS rats, whereas retroperitoneal, omental, and total abdominal fats were unchanged compared to H rats. Whole linseed did not change the individual fat pads in HW rats compared to H rats, whereas defatted linseed lowered retroperitoneal, epididymal, and total abdominal fat in HD rats compared to H rats (Table 1). Liver wet weights were higher in H rats than in C rats. Whole linseed, defatted linseed, and SDG treatment did not change liver wet weight compared to C or H rats (Table 1).

### 3.3. Metabolic Parameters

During oral glucose tolerance test, H rats showed higher basal blood glucose concentrations than C rats. Similarly, H rats showed higher 120 min glucose concentration (Figure 1B). Area under the curve for glucose tolerance test was higher in H rats compared to C, CW, CD, and CS rats. HW, HD, and HS rats were similar to H rats in area under the curve for glucose tolerance test (Table 2). Plasma total cholesterol concentrations were not different among all groups (Table 2). Plasma nonesterified fatty acids and triglyceride concentrations were higher in H rats compared to C rats, and these were unchanged with whole linseed, defatted linseed, or SDG treatment in any of the groups compared to their diet respective controls (Table 2). Plasma insulin concentrations were higher in H rats compared to C rats. HW, HD, and HS rats had lower plasma insulin concentrations compared to H rats, whereas plasma insulin concentrations were higher in CW, CD, and CS rats compared to C rats (Table 2). Plasma leptin concentrations were higher in H rats compared to C rats. CW, CD, and CS rats had no change in plasma leptin concentrations compared to C rats. Whole linseed did not change plasma leptin concentrations, whereas both SDG and defatted linseed decreased plasma leptin concentrations (Table 2).

### 3.4. Cardiovascular, Liver, and Gut Parameters

Heart wet weights were higher in H rats compared to C rats. CW, CD, and CS rats showed no difference in heart weight compared to C rats. HW rats had higher heart weight compared to H rats, whereas HS and HD rats had similar heart weight to H rats (Table 2). H rats had higher systolic blood pressure than C rats. Whole linseed, defatted linseed, and SDG reduced blood pressure in HW, HD, and HS rats, respectively, compared to H rats, whereas these interventions did not change systolic blood pressure in CW, CD, and CS rats compared to C rats (Table 2). H rats had higher ventricular diastolic stiffness than C rats. SDG and defatted linseed reduced diastolic stiffness in HS and HD rats, respectively, compared to H rats, whereas none of the interventions reduced diastolic stiffness in CW, CD, or CS rats compared to C rats (Table 2).

**Figure 2:**
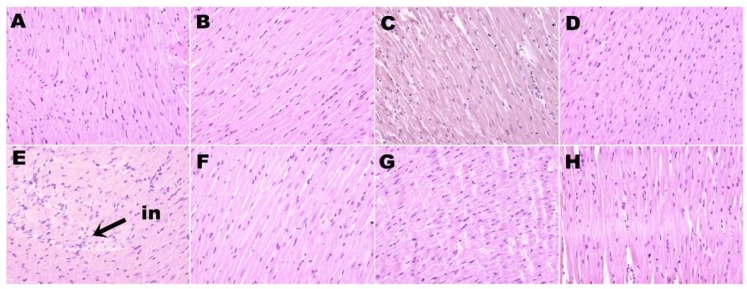
Hematoxylin and eosin staining of left ventricle showing infiltration of inflammatory cells (magnification ×20; shown by arrow) in rats fed corn starch diet-fed rats (**A**), corn starch diet-fed rats treated with whole linseed (**B**), corn starch diet-fed rats treated with defatted linseed (**C**), corn starch diet-fed rats treated with SDG (**D**), high-carbohydrate, high-fat diet-fed rats (**E**), high-carbohydrate, high-fat diet-fed rats treated with whole linseed (**F**), high-carbohydrate, high-fat diet-fed rats treated with defatted linseed (**G**), and high-carbohydrate, high-fat diet-fed rats treated with SDG (**H**). Inflammatory cells are marked as “in”.

**Figure 3:**
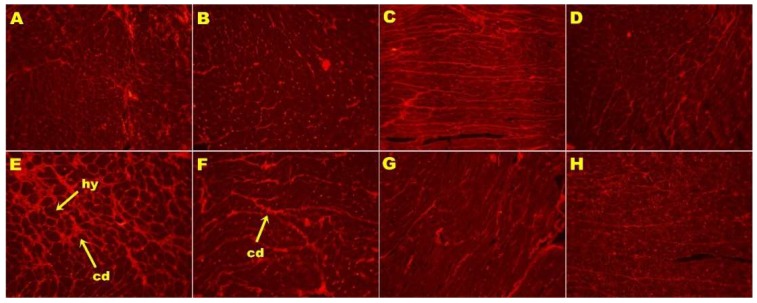
Picrosirius red staining of left ventricular interstitial collagen deposition (magnification ×20; shown by arrows) in rats fed corn starch diet-fed rats (**A**), corn starch diet-fed rats treated with whole linseed (**B**), corn starch diet-fed rats treated with defatted linseed (**C**), corn starch diet-fed rats treated with SDG (**D**), high-carbohydrate, high-fat diet-fed rats (**E**), high-carbohydrate, high-fat diet-fed rats treated with whole linseed (**F**), high-carbohydrate, high-fat diet-fed rats treated with defatted linseed (**G**), and high-carbohydrate, high-fat diet-fed rats treated with SDG (**H**). Collagen deposition is marked as “cd” and hypertrophied cardiomyocytes are marked as “hy”.

H rats showed increased infiltration of inflammatory cells (Figure 2E) and greater interstitial collagen deposition (Figure 3E) as compared to other groups (Figure 2; Figure 3; Table 2). HW, HD, and HS rats had reduced infiltration of inflammatory cells (Figure 2F–H) and ventricular collagen deposition (Figure 3F–H; Table 2) compared to H rats.

**Figure 4:**
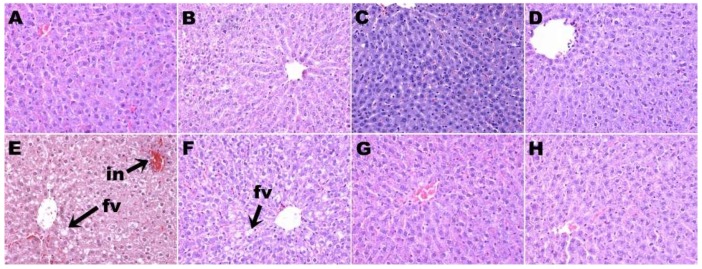
Hematoxylin and eosin staining of liver showing fat vacuoles and infiltration of inflammatory cells (magnification ×20; shown by arrows) in corn starch diet-fed rats (**A**), corn starch diet-fed rats treated with whole linseed (**B**), corn starch diet-fed rats treated with defatted linseed (**C**), corn starch diet-fed rats treated with SDG (**D**), high-carbohydrate, high-fat diet-fed rats (**E**), high-carbohydrate, high-fat diet-fed rats treated with whole linseed (**F**), high-carbohydrate, high-fat diet-fed rats treated with defatted linseed (**G**), and high-carbohydrate, high-fat diet-fed rats treated with SDG (**H**). Fat vacuoles are marked as “fv” and inflammatory cells are marked as “in”.

H rats had higher plasma alanine transaminase activity than C rats. None of the treatments in this study changed the plasma activities of alanine transaminase or aspartate transaminase (Table 2). Staining of liver sections showed increased lipid deposition and inflammatory cell infiltration in H rats (Figure 4E) compared to C rats (Figure 4A). HW (Figure 4F), HD (Figure 4G), and HS (Figure 4H) rats showed decreased inflammatory cell infiltration compared to H rats. HW rats showed some reduction in liver lipid deposition (Figure 4F), whereas HD (Figure 4G) and HS (Figure 4H) rats showed minimal lipid deposition (Table 2). CW (Figure 4B), CS (Figure 4C), and CD (Figure 4D) rats showed no changes in the liver in inflammatory cell infiltration and lipid deposition compared to C rats (Figure 4A; Table 2).

**Figure 5:**
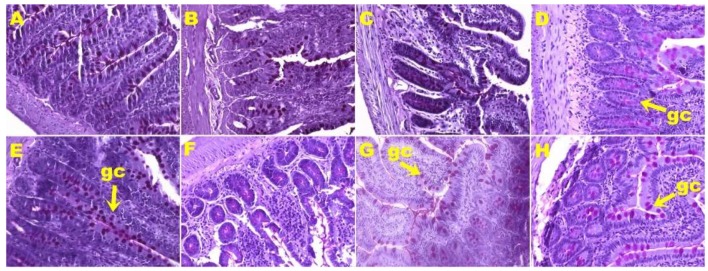
Periodic acid-Schiff staining of ileum showing goblet cells (magnification ×20; shown by arrows) in corn starch diet-fed rats (**A**), corn starch diet-fed rats treated with whole linseed (**B**), corn starch diet-fed rats treated with defatted linseed (**C**), corn starch diet-fed rats treated with SDG (**D**), high-carbohydrate, high-fat diet-fed rats (**E**), high-carbohydrate, high-fat diet-fed rats treated with whole linseed (**F**), high-carbohydrate, high-fat diet-fed rats treated with defatted linseed (**G**), and high-carbohydrate, high-fat diet-fed rats treated with SDG (**H**). Goblet cells are marked as “gc”.

**Figure 6:**
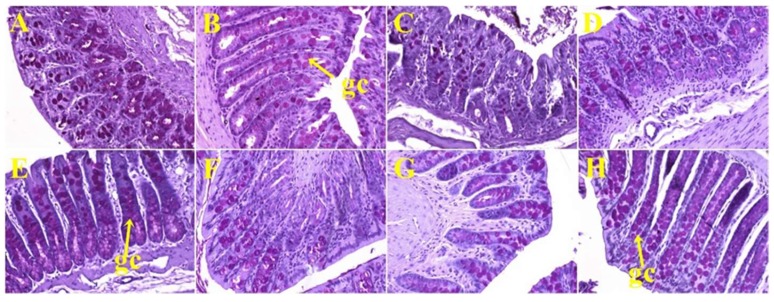
Periodic acid-Schiff staining of colon showing goblet cells (magnification ×20; shown by arrows) in corn starch diet-fed rats (**A**), corn starch diet-fed rats treated with whole linseed (**B**), corn starch diet-fed rats treated with defatted linseed (**C**), corn starch diet-fed rats treated with SDG (**D**), high-carbohydrate, high-fat diet-fed rats (**E**), high-carbohydrate, high-fat diet-fed rats treated with whole linseed (**F**), high-carbohydrate, high-fat diet-fed rats treated with defatted linseed (**G**), and high-carbohydrate, high-fat diet-fed rats treated with SDG (**H**). Goblet cells are marked as “gc”.

Histological analyses of small intestine showed more goblet cells in H rats (Figure 5E) compared to C rats (Figure 5A; Table 2). HW (Figure 5F) and HS (Figure 5H) rats showed no change in the number of goblet cells in the small intestine, whereas HD rats (Figure 5G) showed reduction in the number of goblet cells (Table 2) compared to H rats (Figure 5E). Colon from C rats (Figure 6A) and H rats (Figure 6E) showed no difference in the number of goblet cells (Table 2). HW (Figure 6F), HD (Figure 6G), and HS (Figure 6H) rats showed an increase in the number of goblet cells in colons compared to H rats (Figure 6E; Table 2).

## 4. Discussion

Metabolic syndrome, including obesity, hypertension, impaired glucose tolerance, insulin resistance, dyslipidemia, and fatty liver, is a major risk factor for cardiovascular disease and type 2 diabetes, and may be attenuated by functional foods [18]. Many trials have been conducted to determine the responses to linseed and its components in humans with obesity, hypertension, or diabetes [19,20]. However, few trials have compared responses to individual components of linseed in patients with metabolic syndrome or in appropriate rat models. Rats fed a diet with increased content of fructose, sucrose, and saturated and *trans* fatty acids developed signs of metabolic syndrome in humans, especially abdominal obesity, hypertension, impaired glucose and leptin, dyslipidemia, and diminished cardiac function [13,14,15]. Using this rat model of diet-induced metabolic syndrome, we have now compared responses to whole linseed, defatted linseed flour, and SDG and included comparison with an earlier study on ALA from linseed oil with the same rat model [14].

Our results show that addition of defatted linseed or SDG improved metabolic parameters and the structure and function of the heart and liver, as we previously showed with linseed oil [14]. In contrast, the only metabolic parameter to be improved by whole linseed was plasma insulin concentration, while body weight, abdominal fat pads, and liver parameters were unchanged. Although whole linseed decreased systolic blood pressure, left ventricular diastolic stiffness, infiltration of inflammatory cells, and collagen deposition in the heart, these changes were to a lesser extent than defatted linseed, showing reduced or absent responses in cardiovascular, hepatic structure and function, adiposity, lipid, and glucose parameters. We suggest that the reason for these reduced responses to whole linseed is that the oral bioavailability of ALA, fiber, and SDG when presented as whole linseed is reduced, leading to reduced responses to the whole seeds, even though the components are effective when given individually. This could be tested in further studies by measurement of the pharmacokinetics of linseed components such as ALA, fiber, or SDG in rats fed an obesogenic diet.

In this study with whole linseeds and their components in rats, we were unable to show decreases in body weight or abdominal circumference with whole linseed treatment in diet-induced obese rats. In contrast, we showed decreased total fat and abdominal fat in rats treated with defatted linseed or SDG. We have previously shown that ALA from linseed decreased obesity in the same diet-induced rat model of metabolic syndrome [14]. Other studies also showed decreased obesity with linseed products: young rats fed a linseed flour intervention for the first 90 days showed higher lean mass, lower fat mas, and a smaller adipocyte area [21] and linseed dietary fiber reduced apparent energy and fat digestibility leading to decreased abdominal fat and body weight [22]. Linseed contains 20 to 30% globulin-rich proteins with a high content of arginine [23,24] which has been associated with increases in lean mass [25,26] thereby providing a possible mechanism for lean mass increase as well as fat mass decrease [27]. In high-fat diet-fed mice, SDG decreased abdominal fat and body weight by inducing adiponectin expression at a much higher dose of 0.5 or 1% in diet [28] and inhibiting adipogenesis at a dose of 50 mg/kg/day [29]. Furthermore, the SDG metabolites, enterolactone and enterodiol, induced adiponectin expression, adipogenesis, and lipid uptake in 3T3-L1 adipocytes [28,30]. We are not aware of any studies with whole linseeds in obese rats, but freshly ground flaxseed did not change body weight or blood pressure in non-obese WKY or SHR rats and in cyclosporine-induced hypertensive rats [31,32].

These rodent results translate to some extent to humans. Linseed products reduced human obesity in randomized controlled trials, shown by a meta-analysis of 45 of these trials with 2561 subjects aged 25.6–67.0 years including 21 trials on milled or ground linseed, one on defatted linseed, 18 on linseed oil, and five on linseed lignan but none on whole linseeds [19]. This meta-analysis showed that supplementation of linseed products for more than 12 weeks in individuals with a body mass index higher than 27 kg/m^2^ reduced body weight by an average of 0.99 kg, body mass index by an average of 0.30 kg/m^2^, and waist circumference by an average of 0.80 cm [19]. These changes are relatively small, approximating 1% of these parameters in a 1.80 m tall person weighing 88 kg to give a body mass index of 27 kg/m^2^ fitting the definition of obesity with waist circumference more than 94 cm. Differences of around 1% as in the above meta-analysis on human trials would not be statistically significant in our group of 12 rats treated for eight weeks. In addition, data from the US National Health and Nutrition Examination Survey 2001-10 provided epidemiological evidence that urinary enterolactone is inversely associated with obesity in adult males [33].

Linseed products also reduced blood pressure in rodents. Both linseed oil and SDG prevented the increase in systolic blood pressure in rats with metabolic syndrome induced by feeding with 30% fructose, likely due to decreased oxidative stress [34]. In deoxycorticosterone acetate (DOCA)-salt hypertensive rats, linseed lignan concentrate lowered blood pressure, and improved antioxidant status, serum electrolytes, and lipid profiles [35]. A lignan-enriched linseed powder reduced blood pressure, body weight, and fat accumulation, and improved lipid profiles in rats fed a high-fat and high-fructose diet [36]. In humans with peripheral artery disease, ground linseed (30 g/day) for 12 months decreased central systolic and diastolic blood pressure by 10 and 6 mmHg, respectively, with corresponding changes in plasma oxylipins [37]. Meta-analysis of 15 randomized controlled trials with linseed components on hypertension have shown reductions in both systolic and diastolic blood pressure of 3.10 and 2.62 mmHg, respectively, in a subset of trials of 12 weeks or longer, but there were no effects with linseed oil or SDG on systolic blood pressure [20].

Hyperlipidemia is a key component of metabolic syndrome. In rats fed a high-fat diet, a lignan-enriched linseed powder improved the plasma lipid profile as well as decreasing body weight, visceral fat accumulation, and blood pressure [36]. In rats fed with lard and cholic acid, treatment with powdered linseed or defatted linseed for eight weeks did not change plasma cholesterol, low-density lipoproteins (LDL) cholesterol, or triglyceride concentrations but decreased liver fat and cholesterol and increased bacterial glycolytic activity in the distal intestine [38]. Intervention with linseed powder (30 g/day for 40 days) produced small but significant decreases in body weight and decreased plasma cholesterol, LDL, and triglyceride concentrations in 35 hyperlipidemic subjects [39]. Furthermore, linseed may reduce plasma concentrations of the inflammatory marker, C-reactive protein, in subjects with a body mass index (BMI) > 30 kg/m^2^ [40]; this meta-analysis included trials on ground linseed, flour, oil, and ALA-enriched products, but there were no studies on whole linseeds.

The combination of ALA, dietary fiber, and lignans in linseed may be useful in preventing and treating diabetes, especially in rodent models [41]. SDG and its enteric metabolite enterodiol affected glucose transport and adipogenesis by regulating the transcription of adiponectin, leptin, and peroxisome proliferator-activated receptor gamma (PPAR*γ*) genes [28]. By altering the expression profile of adiponectin and leptin, SDG may increase rates of fatty acid oxidation and mediate an insulin-sensitizing effect [42]. Although these findings are consistent with the decrease in plasma insulin and leptin observed in our study, it is not clear why glucose tolerance was only improved in the whole linseed group.

Thus, our results are broadly consistent with both rodent and human studies on individual signs of metabolic syndrome and individual components of linseeds. However, these literature studies do not compare results in the range of signs in metabolic syndrome in the same subject groups or rodent models, nor allow comparison of linseed components. Furthermore, no study has administered whole linseeds, so the comparison between whole linseeds and components has not been previously made.

Gastrointestinal changes could play a role in the metabolic responses to ground or defatted linseed, but studies on whole linseeds have not been reported. Linseed is a rich source of dietary fiber (35–45%) consisting of soluble and insoluble fiber in ratios that vary between 1:4 and 2:3 [43]. Rats on a control diet fed 10% dietary fiber from linseeds showed decreased body weight with decreased fat digestibility, which was greater when the proportion of viscous dietary fiber was increased [22]. In obese rats fed a high-fat diet with cholic acid, ground linseed prevented an increase in intestinal glucosidase activity while defatted ground linseed increased mucosal disaccharidase activities; both forms decreased fat absorption but only the defatted product decreased liver expression of PPAR*α*, showing important differences between defatted and whole ground linseed [38]. Fermentation of dietary fiber by colonic microflora generates short-chain fatty acids such as acetate, propionate, and butyrate which decrease signs of metabolic syndrome and other gastrointestinal disorders [44]. High-fermentable fiber of milled whole linseed led to increased *Enterobacteriaceae* diversity in mice which was associated with an increased body weight compared to milled defatted linseed [45]. In healthy, non-obese adult men given 0.3 g/kg/day ground linseed for one week, enterolignan production was increased, but there were no changes in fecal metabolome or dominant bacterial communities [46]. Thus, the responses to defatted linseed in contrast to whole linseed could be produced by the increased bioavailable fiber content acting on the colonic microflora and liver, and possibly on goblet cell function. Goblet cells in the gastrointestinal tract are responsible for secretion of mucus, but the location of the goblet cells determines whether secretion is continuous or upon stimulation to form the protective inner colonic mucosal layer [47]. Mice fed a high-fat diet showed increased goblet cells in the duodenum [48]. Dietary intervention with whole ground linseed increased goblet cells, mucus secretion, and concentrations of short-chain fatty acids in healthy male mice which should be beneficial [49]. This increase could be due to an increase in fiber [50], consistent with our results in rats fed defatted linseed. However, the intervention with whole ground linseed worsened the damage by dextran sodium sulfate suggesting a role for context in interventions [49], but similar studies in high-fat diet-fed rats are not available. The different results in rats fed whole linseed, SDG, and defatted linseed suggest that these components of linseed produce different responses in goblet cells in the colon, possibly due to different types of goblet cells, requiring detailed research to define adequately.

The marked differences in responses between whole linseeds and the components of linseeds could be due to toxic compounds present in the whole linseed, such as the cyanogenic glycosides, leading to cyanide production by the activity of bacterial glucosidases in the large intestine [51]. However, ingestion of 30 g linseed by humans produced small and transient increases in plasma thiocyanate concentrations, indicating a low bioavailability of the cyanide from cyanogenic glycosides such as linustatin [52]. Thus, we suggest that a more likely explanation for the lower responses in whole linseeds is a markedly reduced oral bioavailability of the bioactive components when whole linseeds are given.

## 5. Conclusions

This study has highlighted the importance of using a single animal model to investigate the bioactivities of individual functional foods contained in linseed. We hypothesized that whole linseeds and the isolated components would improve cardiovascular, metabolic, and liver changes. We showed that the responses to the whole linseeds were reduced compared to defatted linseed, SDG, and ALA. We suggest that a markedly reduced bioavailability of these components from the whole linseeds underlies the reduced responses. Thus, our hypothesis was substantiated by measurements of physiological responses to the components of linseeds. However, ALA, defatted linseed, or SDG are likely to be better therapeutic agents in metabolic syndrome than whole linseeds.

## Figures and Tables

**Table 1:** Dietary intakes, body composition, and organ wet weights.

Variables	C	CW	CD	CS	H	HW	HD	HS	*p* Value		
									Diet	Treatment	Interaction
Food intake, g/day	35.0 ± 0.6 ^a^	34.6 ± 1.2 ^a^	33.9 ± 1.2 ^a^	35.5 ± 0.8 ^a^	25.2 ± 0.6 ^b^	23.4 ± 0.6 ^b^	24.4 ± 0.4 ^b^	26.3 ± 0.4 ^b^	<0.0001	0.06	0.59
Water intake, mL/day	28.7 ± 1.8 ^a^	30.7 ± 2.1 ^a^	32.7 ± 0.9 ^a^	30.4 ± 2.7 ^a^	25.6 ± 1.0 ^b^	26.9 ± 0.4 ^ab^	25.7 ± 1.1 ^b^	25.2 ± 1.0 ^b^	<0.0001	0.54	0.61
Energy intake, kJ/day	392 ± 7 ^b^	433 ± 14 ^b^	397 ± 14 ^b^	420 ± 13 ^b^	552 ± 13 ^a^	561 ± 14 ^a^	542 ± 9 ^a^	555 ± 8 ^a^	<0.0001	0.11	0.37
Body weight gained (week 8–16), %	7.4 ± 1.0 ^d^	18.2 ± 0.8 ^b^	13.2 ± 1.6 ^c^	9.1 ± 1.7 ^d^	23.3 ± 0.9 ^a^	23.4 ± 0.6 ^a^	22.7 ± 1.4 ^a^	18.9 ± 1.3 ^b^	<0.0001	<0.0001	0.0006
Final body weight (week 16)	375 ± 4 ^a^	432 ± 6 ^b^	400 ± 5 ^a^	392 ± 10 ^a^	526 ± 10 ^c^	539 ± 10 ^c^	521 ± 8 ^c^	519 ± 11 ^c^	< 0.0001	0.0004	0.06
Feed conversion efficiency, %	1.8 ± 0.2 ^c^	4.9 ± 0.3 ^b^	3.2 ± 0.4 ^c^	2.6 ± 0.5 ^c^	9.6 ± 0.6 ^a^	9.2 ± 0.6 ^a^	9.0 ± 0.6 ^a^	8.1 ± 0.7 ^a^	<0.0001	0.004	0.005
Bone mineral content, g	12.0 ± 0.4 ^c^	12.1 ± 0.2 ^c^	11.9 ± 0.2 ^c^	13.2 ± 0.6 ^c^	16.5 ± 0.4 ^a^	17.3 ± 0.7 ^a^	15.3 ± 0.6 ^b^	16.8 ± 0.4 ^a^	<0.0001	0.024	0.20
Total fat mass, g	98 ± 16 ^c^	103 ± 7 ^c^	77 ± 6 ^c^	122 ± 16 ^c^	256 ± 21 ^a^	253 ± 21 ^a^	189 ± 22 ^b^	227 ± 7 ^ab^	<0.0001	0.014	0.25
Total lean mass, g	276 ± 14 ^ab^	317 ± 7 ^a^	309 ± 5 ^a^	267 ± 9 ^b^	268 ± 14 ^b^	277 ± 11 ^ab^	312 ± 15 ^a^	246 ± 7 ^b^	0.035	<0.0001	0.24
Abdominal circumference, cm	18.4 ± 0.2 ^c^	19.1 ± 0.2 ^c^	19.1 ± 0.1 ^c^	18.9 ± 0.2 ^c^	23.0 ± 0.2 ^a^	23.6 ± 0.4 ^a^	21.2 ± 0.2 ^b^	22.7 ± 0.2 ^a^	<0.0001	0.016	0.19
Visceral adiposity index, %	4.9 ± 0.4 ^d^	4.7 ± 0.3 ^d^	4.2 ± 0.1 ^d^	4.8 ± 0.6 ^d^	9.6 ± 0.7 ^ab^	10.4 ± 0.9 ^a^	7.3 ± 0.5 ^c^	8.5 ± 0.4 ^bc^	<0.0001	0.007	0.09
Retroperitoneal fat, mg/mm *	189 ± 18 ^c^	179 ± 18 ^c^	151 ± 20 ^c^	194 ± 31 ^c^	531 ± 41 ^a^	554 ± 59 ^a^	407 ± 31 ^b^	469 ± 31 ^ab^	<0.0001	0.55	0.34
Epididymal fat, mg/mm *	89 ± 10 ^d^	98 ± 8 ^d^	74 ± 5 ^d^	112 ± 20 ^cd^	259 ± 17 ^a^	278 ± 28 ^a^	154 ± 16 ^c^	211 ± 11 ^b^	<0.0001	0.29	0.037
Omental fat, mg/mm *	114 ± 15 ^c^	124 ± 9 ^c^	102 ± 7 ^c^	102 ± 13 ^c^	240 ± 25 ^ab^	280 ± 18 ^a^	198 ± 14 ^b^	207 ± 8 ^b^	<0.0001	0.013	0.26
Total abdominal fat, mg/mm *	392 ± 39 ^d^	401 ± 35 ^d^	308 ± 36 ^d^	408 ± 63 ^d^	1031 ± 79 ^ab^	1113 ± 105 ^a^	683 ± 92 ^c^	887 ± 45 ^b^	<0.0001	0.23	0.18
Liver, mg/mm *	201 ± 5 ^b^	213 ± 12 ^b^	216 ± 7 ^b^	213 ± 8 ^b^	327 ± 12 ^a^	337 ± 15 ^a^	310 ± 8 ^a^	306 ± 9 ^a^	<0.0001	0.32	0.22

Values are expressed as mean ± SEM, *n* = 8–12. Means with different superscripts (a, b, c, or d) differ, *p* < 0.05. C, corn starch diet-fed rats; CW, corn starch diet-fed rats treated with whole linseed; CD, corn starch diet-fed rats treated with defatted linseed; CS, corn starch diet-fed rats treated with secoisolariciresinol diglucoside (SDG); H, high-carbohydrate, high-fat diet-fed rats; HW, high-carbohydrate, high-fat diet-fed rats treated with whole linseed; HD, high-carbohydrate, high-fat diet-fed rats treated with defatted linseed; and HS, high-carbohydrate, high-fat diet-fed rats treated with SDG. * Denotes the values that were normalized against tibial length and presented as tissue weight in mg/mm of tibial length.

**Table 2:** Metabolic, cardiovascular, and liver parameters.

Variables	C	CW	CD	CS	H	HW	HD	HS	*p* Value		
									Diet	Treatment	Interaction
Area under the curve, mmol/L×min	636 ± 19 ^bc^	625 ± 14 ^c^	680 ± 14 ^b^	687 ± 20 ^b^	794 ± 13 ^a^	735 ± 17 ^ab^	770 ± 17 ^a^	765 ± 25 ^a^	<0.0001	0.037	0.09
Plasma total cholesterol, mmol/L	1.5 ± 0.05 ^a^	1.3 ± 0.10 ^a^	1.4 ± 0.10 ^a^	1.6 ± 0.06 ^a^	1.6 ± 0.04 ^a^	1.5 ± 0.10 ^a^	1.6 ± 0.10 ^a^	1.6 ± 0.06 ^a^	0.09	0.023	0.39
Plasma nonesterified fatty acids, mmol/L	0.9 ± 0.2 ^b^	1.5 ± 0.2 ^b^	1.5 ± 0.2 ^b^	1.4 ± 0.2 ^b^	4.3 ± 0.6 ^a^	4.0 ± 0.3 ^a^	3.7 ± 0.4 ^a^	3.7 ± 0.5 ^a^	<0.0001	0.86	0.31
Plasma triglycerides, mmol/L	0.4 ± 0.06 ^b^	0.4 ± 0.10 ^b^	0.5 ± 0.01 ^b^	0.4 ± 0.06 ^b^	1.6 ± 0.30 ^a^	1.5 ± 0.20 ^a^	1.3 ± 0.20 ^a^	1.3 ± 0.20 ^a^	<0.0001	0.68	0.68
Plasma insulin, µmol/L	1.3 ± 0.01 ^e^	2.9 ± 0.05 ^c^	1.7 ± 0.05 ^d^	1.7 ± 0.01 ^d^	7.7 ± 0.09 ^a^	5.9 ± 0.12 ^b^	5.8 ± 0.04 ^b^	6.0 ± 0.11 ^b^	<0.0001	<0.0001	<0.0001
Plasma leptin, µmol/L	3.2 ± 0.40 ^d^	4.2 ± 0.06 ^d^	3.9 ± 0.03 ^d^	2.1 ± 0.62 ^d^	12.3 ± 1.54 ^a^	13.1 ± 0.03 ^a^	10.2 ± 0.08 ^b^	6.3 ± 0.97 ^c^	<0.0001	<0.0001	0.005
Heart, mg/mm *	22.0 ± 1.0 ^c^	25.4 ± 0.7 ^bc^	25.3 ± 0.9 ^bc^	21.5 ± 0.4 ^c^	26.8 ± 1.1 ^b^	31.5 ± 2.1 ^a^	26.9 ± 0.9 ^b^	23.6 ± 0.9 ^bc^	<0.0001	<0.0001	0.18
Left ventricle + septum, mg/mm *	18.1 ± 0.7 ^b^	21.6 ± 0.6 ^b^	20.8 ± 0.8 ^b^	17.2 ± 0.3 ^b^	20.8 ± 0.8 ^b^	27.1 ± 1.8 ^a^	22.3 ± 0.8 ^b^	18.5 ± 0.8 ^b^	0.0002	<0.0001	0.09
Right ventricle, mg/mm *	4.0 ± 0.4	4.5 ± 0.4	4.5 ± 0.3	4.4 ± 0.2	6.3 ± 1.7	5.8 ± 0.4	4.7 ± 0.2	5.0 ± 0.1	0.03	0.79	0.53
Systolic blood pressure, mmHg	128 ± 3 ^bc^	129 ± 2 ^bc^	120 ± 3 ^c^	133 ± 2 ^b^	148 ± 2 ^a^	134 ± 2 ^b^	129 ± 5 ^bc^	133 ± 3 ^b^	<0.0001	0.026	0.0004
Diastolic stiffness constant (*κ*)	23.0 ± 0.4 ^bc^	24.2 ± 0.7 ^abc^	21.9 ± 0.3 ^c^	23.0 ± 0.8 ^bc^	26.6 ± 0.9 ^a^	25.1 ± 0.5 ^ab^	23.7 ± 1.3 ^bc^	22.0 ± 0.5 ^c^	0.034	0.001	0.004
Plasma alanine transaminase, U/L	26.8 ± 2.5 ^b^	30.9 ± 3.8 ^b^	28.3 ± 2.0 ^b^	23.2 ± 1.6 ^b^	33.7 ± 1.5 ^a^	27.8 ± 2.5 ^ab^	33.8 ± 1.5 ^a^	33.6 ± 1.7 ^a^	0.017	0.74	0.017
Plasma aspartate transaminase, U/L	64.6 ± 4.6 ^a^	66.4 ± 5.9 ^a^	66.3 ± 2.8 ^a^	61.6 ± 1.8 ^a^	68.8 ± 2.6 ^a^	61.6 ± 3.7 ^a^	67.5 ± 2.9 ^a^	69.6 ± 6.8 ^a^	0.52	0.84	0.38
Left ventricle collagen deposition, %	6.51 ± 1.0 ^b^	6.57 ± 1.1 ^b^	5.29 ± 0.2 ^b^	4.96 ± 0.5 ^b^	16.1 ± 3.7 ^a^	13.9 ± 1.3 ^a^	8.2 ± 0.5 ^b^	8.3 ± 1.0 ^b^	<0.0001	<0.0001	<0.0001
Left ventricle inflammatory cells, *n*	9.0 ± 2.5 ^b^	7.5 ± 1.2 ^b^	10.5 ± 1.6 ^b^	12.3 ± 1.1 ^b^	60.0 ± 4.0 ^a^	10.8 ± 1.4 ^b^	12.3 ± 1.1 ^b^	10.8 ± 1.1 ^b^	<0.0001	<0.0001	<0.0001
Liver fat vacuoles, *n*	4.0 ± 0.6 ^c^	10.8 ± 1.6 ^c^	4.0 ± 1.2^c^	3.5 ± 1.0 ^c^	75.0 ± 4.6 ^a^	39.7 ± 4.4 ^b^	3.5 ± 1.0 ^c^	1.5 ± 0.9 ^c^	<0.0001	<0.0001	<0.0001
Ileum goblet cells, *n*	91.5 ± 6.0 ^c^	82.3 ± 6.1 ^d^	107.3 ± 2.1 ^abc^	100.3 ± 2.5 ^cd^	124.3 ± 3.4 ^ab^	118.3 ± 8.6 ^ab^	94.8 ± 3.4 ^bc^	110.8 ± 4.4 ^ab^	0.0005	0.3224	0.0004
Colon goblet cells, *n*	97.5 ± 2.9 ^c^	42 ± 5.1 ^d^	84.5 ± 2.6 ^c^	90.8 ± 5.2 ^c^	78.8 ± 3.9 ^c^	135.3 ± 2.3 ^b^	203.8 ± 14.5 ^a^	131.3 ± 7.3 ^b^	<0.0001	<0.0001	<0.0001

Values are expressed as mean ± SEM, *n* = 8–12. Means with different superscripts (a, b, c, d, or e) differ, *p* < 0.05. C, corn starch diet-fed rats; CW, corn starch diet-fed rats treated with whole linseed; CD, corn starch diet-fed rats treated with defatted linseed; CS, corn starch diet-fed rats treated with secoisolariciresinol diglucoside (SDG); H, high-carbohydrate, high-fat diet-fed rats; HW, high-carbohydrate, high-fat diet-fed rats treated with whole linseed; HD, high-carbohydrate, high-fat diet-fed rats treated with defatted linseed; and HS, high-carbohydrate, high-fat diet-fed rats treated with SDG. For histological scoring, values are expressed as mean ± SEM, *n* = 4. * Denotes the values that were normalized against tibial length and presented as tissue weight in mg/mm of tibial length.
